# Curcumin Downregulates Phosphate Carrier and Protects against Doxorubicin Induced Cardiomyocyte Apoptosis

**DOI:** 10.1155/2016/1980763

**Published:** 2016-04-05

**Authors:** Lu Junkun, Chu Erfu, Hasahya Tony, Li Xin, K. C. Sudeep, Zhang Mingliang, Wang Yanqin, Qi XiangQian

**Affiliations:** ^1^Department of Cardiology Division II, Tianjin Medical University Cardiovascular Institute, TEDA International Cardiovascular Hospital, Tianjin 300070, China; ^2^Department of Cardiology Division II, Jiamusi University, 1st Affiliated Hospital, Jiamusi, Heilongjiang 154003, China; ^3^Laboratory of Cardiovascular Immunology, Institute of Cardiology, Union Hospital, Tongji Medical College, Huazhong University of Science and Technology, Wuhan 430030, China; ^4^Jiamusi University, Jiamusi, Heilongjiang 154003, China

## Abstract

*Aim*. To explore the effects of curcumin on phosphate carrier (PiC) and its role in protection against doxorubicin induced myocyte toxicity.* Methods*. Using H9c2 cell line, the cardiotoxic effect of doxorubicin and its mitigation by curcumin were studied. H9c2 cells were cultured with doxorubicin and/or curcumin at various concentrations. Analysis for apoptosis of H9c2 was done using flow cytometry. Confocal laser scanning microscopy was used to record the fluorescence intensity ratios and to determine the mitochondrial permeability transition pore (MPTP) opening state. Oxidative stress was measured using glutathione level, superoxide dismutase activities, and malondialdehyde content. The effect of doxorubicin and curcumin on PiC gene expression was measured by real-time PCR.* Results*. Curcumin decreased mRNA of PiC and was partly protective against oxidative stress, loss of mitochondrial transmembrane potential, and apoptosis induced by doxorubicin. Interestingly, the effect was not clearly dose dependent and the concentration of 12 mg/L was more efficient than 15 and 10 mg/L.* Conclusion*. Curcumin downregulates PiC and partly protects against doxorubicin induced oxidative stress and myocyte apoptosis.

## 1. Introduction

Doxorubicin is a well-established and highly effective antineoplastic agent used to treat a broad range of cancers. However, severe side effects, especially cardiotoxicity, limit its use [1–3]. The cardiotoxic effects of doxorubicin are thought to be mediated in part via disruption of mitochondrial function, increased opening of the mitochondrial permeability transition pore (MPTP), and cytochrome c release, with resulting myocyte apoptosis [4–6].

The mitochondrial phosphate carrier (PiC) serves as the primary means for mitochondrial phosphate import across the inner membrane [7]. It is hypothesized to have a role in cell death as either a component or a regulator of the mitochondrial permeability transition pore (MPTP) complex. New research has also implicated PiC in release of mitochondrial cytochrome c and caspase dependent apoptosis [8]. Indeed, higher PiC levels are associated with increased apoptosis, while PiC downregulation protects cells against apoptosis [7]. Given that that doxorubicin induced myocyte apoptosis is in part mediated via the MPTP, we investigated whether doxorubicin upregulates PiC and whether this is correlated to cellular apoptosis. Moreover, we examined the effect of curcumin, on PiC expression, and its protective role against doxorubicin induced myocyte apoptosis [9–11].

Curcumin is a phenolic compound present in the turmeric roots. It has been shown to exert a cardioprotective effect through decreased lipid peroxidation, membrane stabilization, and prevention of mitochondrial damage and MPTP opening in catecholamine-induced and doxorubicin-mediated cardiotoxicity [12–15].

## 2. Materials and Methods

### 2.1. Materials

The following materials were used: doxorubicin (CAS number: 25316-40-9) with 98% purity from Kinase Chemicals Limited (China), curcumin (natural) CI-75300 from Tokyo Chemical Industry Co. Ltd. (Japan), and Annexin V/FITC apoptosis detection kit from BD company (USA); Hyclone Dulbecco modified Eagle medium with 4 mmol/L glutamine and 4.5 g/L glucose from Thermo Scientific (China); fetal bovine serum from Tianjin Kang Yuan Biology (China); penicillin and streptomycin, sodium pyruvate, sodium bicarbonate, 0.25% Trypsin with and without EDTA, and PBS from Tianjin Solomen Biotechnology Co. Ltd. (China). 5-Tetrachloro-1,1′,3,3′-tetraethylbenzimidazolylcarbocyanine Iodide (JC-1) Mitochondrial Membrane Potential Assay Kit was purchased from Beyotime Institute of Biotechnology. Cellular Glutathione Peroxidase Assay Kit, SOD Activity Assay Kit, and Lipid Peroxidation MDA Assay Kit were from Nanjing Jiancheng Bioengineering Institute (China). The H9c2 embryonic rat heart derived cells were obtained from the Chinese Academy of Sciences (Shanghai).

### 2.2. Methods

#### 2.2.1. Cell Culture Conditions and Preparation of Drugs

The H9c2 embryonic rat heart derived cells were grown in Dulbecco modified Eagle's medium (DMEM) with 10% (V/V) heat inactivated FBS, penicillin G (100 U/mL), and streptomycin (100 mg/mL) at 37°C in 95% CO_2_ humidified incubator. The medium was changed every day and subcultured when the cell population density reached 70–80% confluence. Cells were seeded at an appropriate density according to the experimental design.

Stock solutions of doxorubicin and curcumin were prepared in dimethyl sulfoxide (DMSO) and stored at −20°C. The stock solution was thawed and diluted in culture medium as required prior to experimentation. The final concentration of the DMSO in the medium was always less than 0.1%.

#### 2.2.2. Measurement of Intracellular Oxidative Stress Level including Lipid Peroxidation (MDA) Production, Glutathione (GSH) Levels, and Superoxide Dismutase (SOD) Activities

H9c2 cells were incubated with the same concentration of doxorubicin and subsequently cotreated with various concentrations of curcumin for 2 h. At the end of the treatment, the cells were harvested and sonicated with phosphate buffer (PBS, pH 6.8) containing 1 mmol phenylmethanesulfonyl fluoride (PMSF) to obtain cell homogenates. The thiobarbituric acid-reactive substances (TBARS) method was used to estimate the cellular malondialdehyde (MDA) levels by measuring the absorbance at 532 nm with a spectrophotometer [16].

With respect to the GPX activity, one unit of GPX activity was defined as the amount of enzyme that oxidized 1 nmol NADPH per minute, as measured by the absorbance at 550 nm [17]. The superoxide dismutase (SOD) activity was determined spectrophotometrically at 412 nm by determining the SOD-mediated decrease in the rate of pyrogallol autoxidation under alkaline conditions [18]. The specific activities of SOD and GPX are expressed in terms of units/mg protein.

#### 2.2.3. Flow Cytometry Detection of Apoptosis

For determination of apoptosis, flow cytometry was done using JC-1 dye, which is a cytofluorimetric, lipophilic cationic dye that can selectively enter into mitochondria and reversibly change color from green to red as the membrane potential increases. In healthy cells with high mitochondrial transmembrane potential, it forms complexes known as J-aggregates with intense red fluorescence. In apoptotic or unhealthy cells, due to the loss of mitochondrial transmembrane potential, JC-1 remains in its monomeric form, which shows only green fluorescence.

H9c2 cells were plated onto 6-well plates at 2 × 10^5^ cells/well in a volume of 2 mL and incubated at 37°C in 95% CO_2_ humidified incubator overnight. The cells were then treated with different concentrations of curcumin with or without doxorubicin. Due to an increased loss of cells during the dyeing process after treatment using IC50 dose of doxorubicin at 2 *μ*mol/L, 1/2 IC50 dose at 1 *μ*mol/L was selected to increase survival of cells and incubated for 24 hours. The cells were then trypsinized using 0.25% Trypsin without EDTA and cells were collected by centrifugation and washed twice with PBS with 2% FBS. The cells were then uniformly suspended in JC-1 dye and allowed to incubate at 37°C in 95% CO_2_ humidified incubator for 20 minutes. The cells were then centrifuged and washed twice in 1x staining liquid and suspended in 350 *μ*L of 1x staining liquid for analysis in BD FACSAria Flow Cytometer (BD, USA).

Analysis of cells after dyeing with JC-1 was done within 30 minutes of application of the dye. Mitochondria containing red JC-1 aggregates in healthy cells were detected in the FL2 channel with a bandpass filter designed to detect PE-A (excitation 540 nm, emission 570 nm), and green JC-1 monomers in apoptotic cells were detectable in the FITC channel (FL1) used for detection of fluorescein (excitation 490 nm, emission 520 nm).

#### 2.2.4. Determination of MPTP Opening State

H9c2 cells were plated onto 6-well plates at 2 × 10^5^ cells/well in a volume of 2 mL and incubated at 37°C in 95% CO_2_ humidified incubator overnight. The cells were then treated with different concentrations of curcumin with or without doxorubicin as mentioned above for flow cytometry. The cells were then incubated for 24 hours. The medium was then replaced with JC-1 dye prepared according to the product manual and allowed to incubate at 37°C in 95% CO_2_ humidified incubator for 20 minutes. After adequate dyeing, the cells were viewed using Olympus FluoView FV1000 Confocal Laser Scanning Microscope. Four areas of each slide were photographed and the ratio of green to red fluorescence in each was recorded representing the ratio of low mitochondrial transmembrane potential state and high mitochondrial transmembrane potential state.

#### 2.2.5. Real-Time PCR Analysis of PiC Gene Expression

The total RNA from the cells in each group was extracted and transcribed into cDNA using random primers reverse transcriptase. The reverse-transcribed cDNA was used as a template for polymerase chain reaction (PCR) amplification using Taq DNA polymerase. The primer sequences used in the present study are listed in Table 1. The following reaction conditions were used: predenaturation at 94°C for 5 min, followed by 40 cycles of denaturation at 95°C for 10 s, annealing at 60°C for 30 s, and extension at 72°C for 30 s. Beta-actin (*β*-actin) was used as an internal control. The relative mRNA expression was quantified through a comparison of the cycle threshold (Ct) values. The experimental data were processed using the 2^−ΔΔ^Ct method: ^ΔΔ^Ct = (Ct target − Ct internal control) experiment group − (Ct target − Ct internal control) normal control group. Each experimental group was analyzed in triplicate.

## 3. Results

### 3.1. Determination of Intracellular Oxidative Stress Level

**Figure 1:**
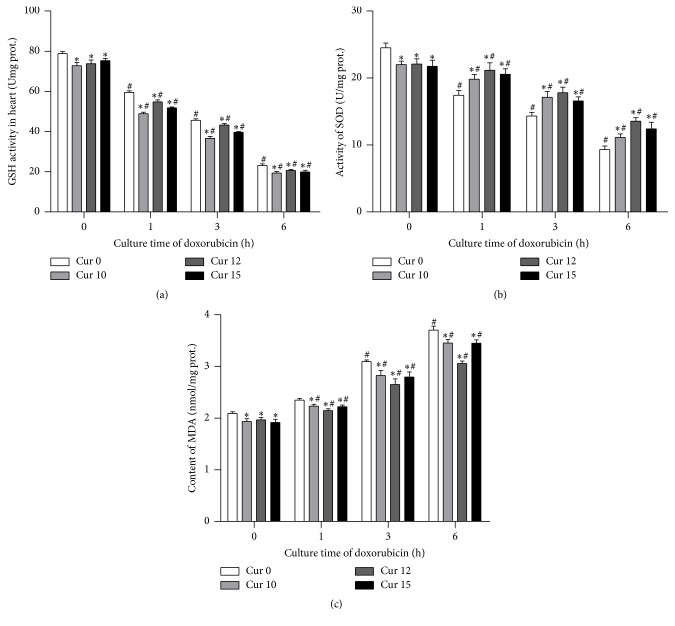
Oxidative stress showed obvious increase with time after addition of doxorubicin and this was alleviated by curcumin. (^#^ *P* < 0.05 versus Negative control (Dox 0 h-Cur 0 min)) (^*∗*^ *P* < 0.05 versus Cur 0, Dox + at each time point). (a) Following culture with doxorubicin, GSH levels declined with time while curcumin obviated this effect at various doses. (b) SOD activity decreased with time after culture with doxorubicin, and this effect was decreased by curcumin at various doses. (c) Doxorubicin culture increased MDA content over time, an effect that was attenuated by curcumin. 12 mg/L was the most efficient dose of curcumin in decreasing MDA content following culture with doxorubicin.

In order to measure oxidative stress induced by doxorubicin in H9c2 cells, we used 3 kits. As anticipated, with increased time of cell culturing with doxorubicin, there was a marked increase in oxidative stress as shown by GSH levels decline, MDA content increase, and SOD activities decrease. The oxidative stress progressively reduced with addition of increasing amounts of curcumin. 12 mg/L dose of curcumin showed the most obvious decline. These results suggest that curcumin partly protects against high oxidative stress states caused by doxorubicin (Figure 1).

### 3.2. Determination of Apoptosis Using Flow Cytometry

We observed a dose dependent decrease in doxorubicin toxicity when H9c2 cells were pretreated with curcumin at the selected low doses (Table 2). Among the three different doses tested, 12 mg/L of curcumin showed the largest decrease in percentage apoptosis which was statistically significant (*P* < 0.05). There was an observed difference in apoptosis between groups treated with 10 and 15 mg curcumin and controls, though this did not reach statistical significance, *P* > 0.05 (Table 2).

### 3.3. Determination of MPTP Opening State Using Confocal Laser Scanning Microscopy

The change in the color of H9c2 cardiomyoblasts in different mitochondrial transmembrane potential states using the JC-1 dyeing process was verified using Olympus FluoView FV1000 Confocal Laser Scanning Microscope. Visualization of H9c2 cells after dyeing with JC-1 using confocal laser scanning microscopy showed healthy cells with intense red fluorescence and apoptotic or unhealthy cells with green fluorescence.

The means were then compared with controls using one-way ANOVA with Dunnett's post hoc test. Results revealed that groups pretreated with 12 and 15 mg/L curcumin prior to doxorubicin treatment had significantly lower ratio between the green and red fluorescence indicating a significant increase in transmembrane potential due to decrease in the opening of MPTP compared to control (Table 3).

### 3.4. Curcumin and Doxorubicin Modulate Expression of Phosphate Carrier Protein

**Figure 2:**
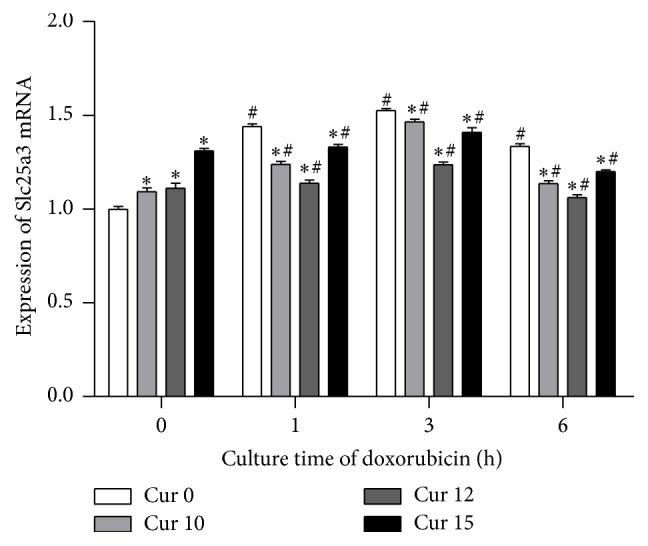
The expression of PiC (Slc25a3) gene was significantly increased by doxorubicin, while curcumin decreased PiC expression. In addition, the PiC in groups of Cur 0 was also significantly increased over time following doxorubicin treatment of the cells. (^*∗*^ *P* < 0.05 versus Cur 0, Dox + at each time point) (^#^ *P* < 0.05 versus negative control (Dox 0 h, Cur 0 min)).

We investigated the effect of doxorubicin and curcumin on PiC gene expression. We found that phosphate carrier gene expression in cardiomyocytes was significantly decreased by curcumin and increased by doxorubicin (Figure 2). At zero hours, we noted an initial increase in PiC following addition of curcumin. This could be due to the fact that curcumin itself, even in lower concentrations, also has some level of toxicity [11], to which the cells may adapt over time. 12 mg/L curcumin was the most effective dose in decreasing PiC.

## 4. Discussion

Although doxorubicin is a widely used chemotherapy agent, its cardiotoxic side effects have limited its use. The mechanism by which doxorubicin causes cardiotoxicity is not fully known. Some studies have implicated the mitochondrial permeability transition pore (MPTP) excessive opening as key to doxorubicin induced apoptosis [19]. Recently, curcumin has been shown to have protective effects against doxorubicin induced cardiac toxicity [9]. Given that the mitochondrial phosphate carrier (Pic) is a key component of MPTP and its upregulation increases apoptosis [8], we explored the possibility that doxorubicin upregulates PiC and that curcumin downregulates PiC and protects against cellular apoptosis.

Flow cytometry results revealed a significant decrease in apoptosis (*P* < 0.05) in cells pretreated for 2 hours with 12 mg/L concentration of curcumin prior to exposure to 1 *μ*mol/L concentration of doxorubicin compared to doxorubicin alone treated group. However, the decrease in apoptosis in cell populations pretreated with 10 and 15 mg/L of curcumin was not statistically significant. This finding was in contrast to a recent study that showed that pretreatment with nontoxic concentrations of curcumin sensitized H9c2 cells to doxorubicin-mediated apoptosis by generation of ROS [11]. Confocal laser scanning microscopy revealed that there was a decrease in loss of mitochondrial transmembrane potential in the groups pretreated with curcumin 12 and 15 mg/L compared to control.

A new finding was that phosphate carrier protein expression was significantly increased with prolonged time of doxorubicin cellular exposure while it was obviously decreased by curcumin. It is likely that curcumin reduces the expression of mitochondrial phosphate carrier and possibly improves the oxidative stress injury of mitochondria, decreases the rate of myocardial cell apoptosis, and thus protects myocardial cells. This may be another mode of action of curcumin protection against anthracycline toxicity, in addition to the known mechanisms such as scavenging or neutralizing of free radicals, inhibition of oxidative enzymes like cytochrome P450, oxygen quenching and making it less available for oxidative reaction, interacting with oxidative cascade and preventing its outcome, and disarming oxidative properties of metal ions such as iron [9]. The protective action of curcumin through reduced oxidative stress in cardiomyocyte cells was also evidenced by our findings of salvaged GSH and SOD activity and a decrease in MDA content (Figure 1).

Our results only open a window, and further research into the mechanisms and role of PiC in curcumin protection against doxorubicin cellular toxicity is warranted.

## 5. Conclusion

Doxorubicin upregulates PiC, while curcumin downregulates PiC and protects against doxorubicin induced myocyte apoptosis.

## Figures and Tables

**Table 1:** Primers used for quantitative real-time RT-PCR.

Gene	Sequence 5′→3′	Temperature profile
PiC	GTGGTTTGGCTAAAGGATGG GGGCAATGTCAGCGAAGAA	Denaturation 95°C, 15 s
		Annealing 60°C, 40 s
		Extension 72°C, 30 s

*β*-actin	CGTAAAGACCTCTATGCCAACA AGCCACCAATCCACACAGAG	Denaturation 95°C, 15 s
		Annealing 60°C, 40 s
		Extension 72°C, 30 s

**Table 2:** Flow cytometry data showing percentage of apoptotic cells after either treatment with doxorubicin alone for 24 hours or pretreatment with curcumin low doses of 10, 12, and 15 mg/L for two hours.

Groups	Mean (%), *n* = 3	Std. deviation
Control	9.2000	0.58310
Cur alone	8.3389	0.99952
Dox alone	29.3667^#^	6.50410
Cur 10 + Dox	23.4667	4.73533
Cur 12 + Dox	**17.6000** ^*∗*^	0.51962
Cur 15 + Dox	22.2333	4.03774

Note: ^*∗*^ *P* < 0.05 versus Dox alone group; ^#^ *P* < 0.05 versus control group.

**Table 3:** Mean values of the ratio of green to red fluorescence indicating the ratio of mitochondrial transmembrane potential state (MPTP opening state) in each group.

Group	Mean	±SD
Control	0.2592	0.02777
Cur alone	0.2350	0.04760
Dox alone	0.8275^#^	0.30977
Cur 10 + Dox	0.6150	0.14572
Cur 12 + Dox	**0.3900** ^*∗*^	0.22559
Cur 15 + Dox	**0.4375** ^*∗*^	0.16432

Note: ^*∗*^ *P* < 0.05 versus Dox alone group; ^#^ *P* < 0.05 versus control group.
